# Gamma-hydroxybutyrate withdrawal syndrome: a case report

**DOI:** 10.1186/1757-1626-2-6530

**Published:** 2009-03-25

**Authors:** Michael A Kuiper, Nicole Peikert, E Christiaan Boerma

**Affiliations:** 1 Department of Intensive Care Medicine, Medical Centre Leeuwarden, PO Box 888, 8901 BR Leeuwarden, The Netherlands, Department of Intensive Care Medicine, Academic Medical Centre Amsterdam Hermes Critical Care Group; 2 Department of Intensive Care Medicine, Medical Centre Leeuwarden, PO Box 888, 8901 BR Leeuwarden, The Netherlands, St. Josef-Hospital, Bonn, Germany; 3 Department of Intensive Care Medicine, Medical Centre Leeuwarden, PO Box 888, 8901 BR Leeuwarden, The Netherlands

## Abstract

**Introduction:**

To raise awareness among health care workers of the risk of withdrawal symptoms after longstanding and intense abuse of gamma-hydroxybutyric acid.

**Case presentation:**

A 23 year old Caucasian woman presented with gamma-hydroxybutyric addiction and withdrawal syndrome. The symptoms of gamma-hydroxybutyric withdrawal in this patient initially went unrecognized, upon which her situation deteriorated in such a way that she needed to be admitted to the Intensive Care Unit for airway protection and mechanical ventilation. Treatment with high doses of benzodiazepines led to liberation of the ventilator and further recovery.

**Conclusion:**

Withdrawal symptoms of gamma-hydroxybutyric addiction are often not well recognized and the responsible physicians at Emergency Department, Intensive Care Unit and the Psychiatry ward need better understanding of diagnose and treatment. Gamma-hydroxybutyric acid withdrawal is potentially life threatening and its management may require a multidisciplinary approach. Early recognition of gamma-hydroxybutyric acid withdrawal may lead to better management of these patients.

## Introduction

The former anaesthetic gamma-hydroxybutyric acid (GHB; sodium oxybate), has developed into a popular and widely used psychoactive party drug. It is easily produced and often sold in bottles as a liquid. GHB can lower inhibitions, create feelings of euphoria, and increase libido. Misuse of GHB may lead to acute loss of consciousness and subsequent visits to the Emergency Department (ED). Death as a result of misuse has been reported [1]. It is less known however that GHB can induce tolerance and dependence and therefore addiction. Cessation of GHB use after prolonged abuse may lead to withdrawal symptoms, [2] and may even be lethal [3]. As there only are few case reports on GHB withdrawal syndrome, this syndrome is not yet well known and therefore difficult to diagnose.

After ingestion, GHB is transformed into the inhibitory neurotransmitter gamma-aminobutyric acid (GABA), [4] with preference for the GABA_b_ receptor. This may explain why its psychotropic effects resemble those of alcohol and benzodiazepines (euphoria, amnesia, drowsiness, reduced anxiety, loss of motor control) and ecstasy (enhanced sensuality, emotional warmth) [5]. GHB also exhibits modulating effects on the dopaminergic systems and it may thus induce extra pyramidal signs. Physical effects are nausea, vomiting, bradycardia, hypothermia, hypernatremia and metabolic alkalosis.

Therapeutically it has been used in anaesthesia. As it has only minimal analgesic effects and the potential to provoke myoclonias, its use in anaesthesia has been abandoned [6]. Another therapeutic effect is to limit the symptoms of alcohol withdrawal [7]. GHB also influences the serotonergic pathways, [8] and it is used to treat sleep disorders, mainly to diminish cataplexy in narcolepsy [9].

Initial GHB withdrawal features are similar to those for alcohol: tachycardia, delirium, hallucinations, paranoid psychosis, autonomic instability, disturbed behaviour, anxiety, tremor, and restlessness [6,10]. As time progresses, choreatic movements of extremities and restlessness of the tongue become prominent, resembling Huntington's disease or tardive dyskinesias. Studying the literature, it becomes evident that there is a large variation in withdrawal symptoms [10]. The symptoms are a result of a GABA-depletion as a consequence of GHB dependence. GHB is rapidly cleared, and dependency can only develop with sustained levels of GHB, which requires intake every 1-3 hours. Acute GHB withdrawal syndrome is difficult to diagnose and may lead to life-threatening complications, as is demonstrated in the following case report.

## Case presentation

A 23-year-old Caucasian woman was presented at the ED of our hospital after having lost her consciousness in a bus. Bystanders reported convulsions during this event. At our ED, the woman was too weak and too confused to answer questions, therefore taking a correct history was not possible. Her medical records showed substance abuse: cocaine, cannabis, amphetamines, crack and GHB. At time of presentation at the ED, she was awake, but with diminished consciousness and incoherent thoughts, which was scored as a Glasgow Coma Score (GCS) of 14. Except for tachycardia, vital signs and electrocardiography were normal.

She was diagnosed with GHB intoxication and admitted to the medical ward for observation because her consciousness was still slightly lowered. The evening of the day of admittance, she became very agitated, had hallucinations and recognized no persons other but her boyfriend. Vital signs remained stable. Lorazepam was started at 1 mg 4 times per day and raised to 1 mg 6 times per day. As the agitation increased, she had to be physically restrained. Being still uncooperative, she received 5 mg haloperidol and 50 mg clorazepate.

The next day she remained disoriented and still had hallucinations. Because of her uncooperative behaviour, she was transferred to the psychiatry ward, next to, but separated from the hospital, where she was treated with clorazepate 50 mg daily, oxazepam 25 mg 3 times per day, biperiden 2 mg 2 times per day and haloperidol in a rising dose up to 5 mg 3 times per day.

Six days later, her psychiatrist sent her back to the ED because of deterioration with loss of motor control and fever. The patient was again very agitated. Physical examination showed no abnormalities and neither did a computer tomography (CT) of the brain. At that time she had a fever of 39.3 °C, a blood pressure 109/76 mmHg, and a pulse rate of 132/min. Pulse oximetry showed an oxygen saturation of 97%, while her respiratory rate was 33/min and she now scored a GCS of 10 out of 15 (E2 M5 V3). Laboratory investigation showed WBC 20.000/μl, C-reactive protein (CRP) 50 mg/dl and a highly increased creatinekinase (CK) of 6000 U/l. Again she was admitted to the medical ward for observation and treatment with lorazepam. The next day, the situation worsened with hypoxia (pO2=8.2 kPa), a rising CK (9800 U/l) and extreme motion restlessness of extremities with choreatic movements and peri-oral hyperkinesias. The choreatic movements, resembling Huntington's disease, prompted us to consider a cause related to the GABA system, leading eventually to the diagnosis of GHB withdrawal syndrome. Bruises were visible on knees and elbows. With the diagnosis of severe GHB withdrawal syndrome and rhabdomyolysis she was admitted to the Intensive Care Unit (ICU). The midazolam (50 mg/h) and clonidine (120 mcg/h) needed to manage the unrest, inhibited patient's coughing reflexes and lead to diminished clearance of secretions of her upper airway, resulting in hypoxia, which necessitated intubation and mechanical ventilation. Even despite these high doses of sedatives, she still sometimes had phases of being awake and sitting upright in bed. Because of rhabdomyolysis, she received 6 L infusion fluid intravenously per 24 hours. There were no signs of renal failure at any time. After three days the neurological situation had improved significantly. The patient was extubated despite having need of high doses of midazolam and clonidine, which could be decreased over days. Temperature, CK, WBC and CRP returned to normal values. Eight days after admission to the ICU, the patient was discharged to the medical ward with oxazepam 80 mg 3 times per day. Soon thereafter she could be discharged from the hospital, without any obvious neurological sequelae.

Her parents later reported that over the last three months for admittance, their daughter was using GHB every two to three hours. Based on information given by her parents and other relatives, our patient daily GHB intake was estimated between 60 and 90 g. Patient later confirmed this.

## Discussion

GHB withdrawal syndrome has been reported in a few case reports. As it is not well known, it may not easily be recognized. The risk of a withdrawal delirium seems to depend on daily dose of ingestion of GHB and duration of withdrawal [10]. Our patient carried a bottle of liquid GHB with a concentration of 500 g/L (50% g/v). Years of use or co-ingestants do not have any influence on the presence or absence of a delirium [10]. Complications of a GHB withdrawal are delirium, rhabdomyolysis with myoglobinuria, and Wernicke's encephalopathy, severe muscle spasms and cardiac arrest [3].

Differential diagnosis of GHB withdrawal includes neuroleptic malignant syndrome, serotonergic syndrome, catatonia, alcohol withdrawal syndrome, delirium tremens, steroid psychosis and acute MDMA intoxication. As GHB influences the dopaminergic pathways, the underlying mechanism of acute GHB withdrawal might be related to neuroleptic malignant syndrome.

For the treatment of a GHB withdrawal, benzodiazepines (diazepam, lorazepam), often in high doses, are recommended [6]. Benzodiazepines are indirect GABA_a_ agonists, by increasing the receptor affinity for GABA [3]. As GHB has a preference for the GABA_b_, this may explain why in cases of severe GHB addiction, even high doses of benzodiazepines will not be sufficient to control withdrawal symptoms. In that event, a combination with anticonvulsants or non-benzodiazepine sedatives (barbiturates, chloral hydrate) is needed [6,7,11]. Clonidine may be used to diminish the sympathetic symptoms as tachycardia and sweating. Severe withdrawal lasts 8 to 10 days [10]. Controversial benefit is reported from butyrophenone anti-psychotics like haloperidol. Glasper et al. report a case of a patient with GHB withdrawal, which was treated with droperidol without any side effects [12]. Other authors describe severe side effects after administration of butyrophenones like haloperidol or droperidol [13]. McDonough et al. proposed an algorithm for the management of GHB withdrawal, [10] as did Rosenberg et al [13].

Our patient showed delirium, cognitive disturbances, catatonia, rhabdomyolysis, choreatic movements, fever and severe agitation. After recognition of the GHB withdrawal syndrome, and use of high doses of benzodiazepines, she ultimately recovered. This confirms previous reports on (treatment of) GHB withdrawal syndrome.

## Conclusion

GHB withdrawal may lead to life-threatening complications, as demonstrated in this case report. Next to other withdrawal symptoms, choreatic movements of limbs and mouth, may be a hint towards the diagnosis. As there are only a few case reports on GHB withdrawal, it is not possible to compare different drug treatment regimes. Because of the high doses of benzodiazepines that are often needed, it may be necessary to a hospitalize patients for treatment, if necessary in the ICU. GHB withdrawal management often will require a multidisciplinary approach, and ICU treatment with mechanical ventilation may be life-saving. Since butyrophenones may worsen the withdrawal symptoms and cause or worsen rhabdomyolysis, these should probably be avoided in the treatment of psychosis caused by GHB withdrawal.

## List of abbreviations

GCS: Glasgow Coma Scale; CK: Creatinekinase; CRP: C-reactive protein; CT: Computer Tomography; ED: Emergency Department; GHB: gamma-hydroxybutyric acid; ICU: Intensive Care Unit; MDMA: 3,4-methylenedioxymethamphetamine; WBC: White blood cells; GABA: gamma-aminobutyric acid.

## Consent

Written informed consent was obtained from the patient for publication of this case report. A copy of the written consent is available for review by the Editor-in-Chief of this journal.

## Competing interests

The authors declare that they have no competing interests.

## Authors' contributions

MK wrote the final manuscript, NP collected all details to provide the case presentation and drafted the first version of the manuscript, CB initially treated the patient and initiated the writing of the case report.
